# Notes and Notices

**Published:** 1892-03

**Authors:** 


### NOTES AND NOTICES.

DR. AMOS J. GIVENS, who was formerly Interne at the New York State Homœopathic Hospital for Insane, at Middletown, N. Y., and later Assistant Physician at the Westboro' Insane Hospital, in Massachusetts, has opened a private Homœopathic Sanitarium, Stamford Hall, Stamford, Conn.

DR. OLIN M. DRAKE has removed from Ellsworth, Maine, to No. 70 Huntington Avenue, Boston, Mass.

DR. SWAN'S POTENCIES.—During the many years I have been making high potencies, I have given them away, or sold them much below the actual cost. I did this in order to induce physicians to use them, knowing that in so doing they could make more speedy and permanent cures, and thus they would be better able to appreciate Homœopathy. I have never allowed any one to assist me in their preparation, and as the demand for the highest potencies is increasing, and taxing my time and strength, in order that I may receive partial compensation for my work in future, I have decided to raise my prices. Hereafter the price of grafts of any potency will be $1.00. But if more are ordered at the same time, the price will be 25 cents for each, except the first. The price of No. 1548 vials in pellets will be $2.00 for the first, and for all others in the same order $1.00 each. For pellets in half-ounce vials, $4.00 for the first, and $2.00 each for all others in the same order. For Potentizing diseased products $5.00, and the six potencies in dilution will be returned. *No order will be filled unless accompanied by the money.* The potencies I have in stock are the 1M, 50M, CM, MM, CMM, DMM. Having selected the material and made the potencies myself, I can vouch for the purity and reliability, and I have yet to hear anything but praise for their effective action. Respectfully, SAMUEL SWAN.

THE YALE SURGICAL CHAIR.—The Canton Surgical and Dental Chair Co., whose advertisement appears in this number, desire us to announce that on the night of January 30th their large factory for the manufacture of their unique and wonderful chair was totally destroyed by fire, entailing a loss of $30,000. They have, however, started to rebuild, and before this announcement shall have been read by the profession will be going on as usual in the manufacture of chairs.

DR. EDWARD RUSHMORE has taken exception to the report of his remarks at the meeting of the I. H. A., as published on page 49 of February number of this journal. The corrected report should read thus: "Microscopic examination has shown the fat globules in the milk of the Ayrshire to be finer than in that of any other breed."

THE KLIP.—Our readers must have noticed in the advertising pages of this journal, from month to month an advertisement of a "Binding Klip" for pamphlets. The editor personally recommends this clever device to every member of the profession as a convenient, cheap, and efficient means of preserving pamphlets in an orderly condition. It has been adopted in our office, and we would not be without it. We learn that it has been adopted by the Boston Public Library, Boston Athenæum, New Haven Public Library, the public libraries of Springfield, Northampton, Brockton, Spencer, Pittsfield, and North Adams, Mass., and Pittsburgh, Pa.; by the libraries and reading rooms of Yale and Amherst Colleges; by the U. S. Department of the Interior, U. S. Agricultural Department U. S. Navy Department, etc.; by *Youth's Companion*, and other papers; by many Y. M. C. A. Reading Rooms; N. Y. State Museum, at Albany, N. Y., and by scores of private men of all occupations

THE LIFE INSURANCE INQUIRY.—The Missouri Institute of Homœopathy has issued a report made by its Secretary, Dr. William P. Cutler, 1227 Michigan Avenue, Kansas City, Missouri, upon the result of the inquiry made to two hundred Life Insurance companies, as to whether they discriminate against homœopathic physicians in selecting their medical examiners of applicants for insurance. Twenty-nine answers only were received, of which twelve claimed to have homœopathic examiners, and gave the names of these examiners; ten claim that they do not discriminate, and one—*The Commercial Travelers' Insurance and Mutual Accident Association*, of Utica, New York, has a homœopathist for its chief medical examiner. The remainder refused to answer or disputed the right to ask such questions.

THE MISSOURI INSTITUTE OF HOMŒOPATHY will hold its next meeting in St. Louis, April 12th, 13th, and 14th. Physicans everywhere are invited to attend, and prepare papers for the meeting. Notice of the titles of the papers should be sent to the General Secretary, William P. Cutler, M. D., 1227 Michigan Avenue, Kansas City, Missouri.

MEDICAL TREATMENT OF SICK ANTS.—The ant is said to have the largest brain according to its size of any creature in the world, and it stands to reason that so much brain must give rise to numerous complaints of the head, and some things we have seen through the microscope recently strengthen us in our opinion.

On one occasion a number of poor, sickly ants came up to the surface, each accompanied by several attendants. I know they were sick because they were so emaciated and feeble—indeed, we imagined a whole hospital had turned out for an airing, but there seemed to be another object. A grave, strong-looking ant was sitting above the ground on a brick wall, and imagine our surprise when an invalid crept slowly up the wall, and immediately the physician ant began to make passes over the afflicted one's head, as though he were trying to effect a cure by the electrical qualities of his antennæ or feelers. The sick one remained perfectly motionless, with bowed head, while going through the operation.

And so one after another came up for treatment, from sunrise until sunset when I ceased observation.

The next morning early we went out again to watch further progress, but the mites were all gone save a very few dead ones that must have been too far gone to be cured. The dead ones were much emaciated indeed.—*Portland Transcript.*

IMPORTANT NOTICE AND REMOVAL.—To avoid failure or doubtful success in use of Peroxide of Hydrogen be sure you get *Marchand's Medicinal;* no substitute can replace it, statements of dealers interested or unscrupulous parties to the contrary notwithstanding. There is great inducement to use substitutes for this article for the reason that Peroxide made for bleaching and varying trade purposes costs to produce only a fraction of what Marchand's Medicinal costs and the unscrupulous druggist or dealer pockets the difference in profit at the expense in reputation of the physician. Marchand's Peroxide of Hydrogen, Medicinal, is put up in 4-oz., 8-oz., and 16-oz. bottles only. Every careful physician should be familiar with the character of the packages in order to frustrate dishonest substitution and assure success. Drevet Manufacturing Company, 28 Prince Street, New York.

RESIDENT PHYSICIAN WANTED.—A competitive examination will be held at the Children's Homœopathic Hospital, Philadelphia, for Resident and Associate Resident Physicians in the early part of April, 1892.

The experience obtained in the Institution and its Out-Patient Department, of which the Residents have charge, is particularly valuable to a graduate. Applicants will please address Dr. Bushrod W. James, President of the Hospital, or Dr. Joseph M. Reeves, President of the Medical Board, 926 North Broad Street, Philadelphia, Pa.

JOURNALS FOR SALE.—Dr. Clarence M. Selfridge, 400½ Haight Street, San Francisco, California, has for sale the following copies of journals belonging to the estate of the late Dr. G. M. Pease: THE HOMŒOPATHIC PHYSICIAN, 1888, complete; 1889, May and September wanting. The Repertory numbers are included in this set. 1891, December number wanting. *The Medical Advance*, 1885, February number, 16 copies; 1887, January number wanting; 1888, 1889, 1890, complete; 1891, July, September, and November numbers wanting.

PRE-HAHNEMANNIAN HOMŒOPATHY.—"Irrespective of one's bias as to the several schools of medicine, it is interesting to note the fact that the poetical mind of Milton anticipated the theory of Hahnemann, as is evinced by the following extract from his preface to *Samson Agonistes.* He remarks that tragedy has power, 'by raising pity or fear or terror, to purge the mind of these and such like passions; that is, to temper and reduce them to just measure with a kind of delight, stirred up by seeing those passions well imitated, nor is nature wanting in her own effects to make good this assertion; for so in physic, things of melancholic hue and quality are used against melancholy, sour against sour, salt to remove salt humors.' I do not remember ever seeing this Miltonic statement of *Similia Similibus Curantur* commented on before." A. S. Hayden. M.D., Columbiana, Ohio.—From the *Argus*, Jan., 1892.

THE HAHNEMANN HOSPITAL.—The Hahnemann Hospital, of Philadelphia, is now fitted out with what is claimed to be the most complete surgical appliances, instruments, and conveniences to be found in any hospital in the country. The instrument room, the surgical supply room, the splint room, and the private operating room have just been finished and furnished at much expense. Things have been arranged in the most aseptic manner, according to the principles of modern surgery.

The amphitheatre, which was, when first opened, considered one of the finest and best arranged in the country, if not in the world, has recently undergone improvements which still further add to its conveniences. Not only is it well lighted by windows on the four sides, but from the roof also. Directly over the operating table is arranged a circlet of electric lights, which, together with a number of side lights, enables the operator to work at night and the students to have a full view of the subject without a shadow being thrown in any direction. The table tops are all of plate glass and the frames of polished metal, thus insuring cleanliness and avoiding all possibility of any disease germs finding a hiding place. The sterilized and other aseptic napkins are kept in air-tight stone jars, free from any contact with deleterious substances.

THE STATE AND MUNICIPAL BOARDS OF HEALTH of the several States held a conference in Chicago, on Thursday, January 14th, 1892, with a view to arranging for an exhibit of the official sanitary work of the country at the World's Columbian Exhibition to be held at Chicago in 1893.

THE COMMITTEE OF THE AMERICAN PUBLIC HEALTH ASSOCIATION "to arrange for the meeting of 1893 in the city of Chicago, and to make it as far as possible an International Congress of Hygiene and Public Health," is as follows: Dr. John H. Rauch, Springfield, Illinois, *Chairman;* Dr. A. N. Bell, Brooklyn, New York; Dr. Samuel W. Abbot, Wakefield, Massachusetts; Dr. Peter H. Bryce, Toronto, Ontario; Dr. Charles N. Hewitt, Red Wing, Minnesota; Dr. Lucien F. Salomon, New Orleans, Louisiana; Dr. H. D. Fraser Charleston, South Carolina.

---

### FUN FOR DOCTORS.

SIR ARTHUR SULLIVAN is credited with saying, in reply to an ignorant, but pretentious woman who asked him if Bach were composing anything nowadays: "No, madam, he is decomposing."

A FAMILY named Boyle has set up a claim to the ownership of much of the territory occupied by Lansing, Mich. It is not strange that Boyles should need Lansing, however.

WHY THEIR BANK ACCOUNT GROWS.—"Patient waiters are never losers," says a very old proverb, and it explains why some doctors eventually get rich.

DID NOT EARN HIS FEE.—Defendant.—"Now, docthor, by vartue of your oath, didn't I say: 'Kill or cure, docthor, I'll give you a guinea?' and didn't you say: 'Kill or cure, I'll take it?'"

Doctor.—"You did; and I agreed to the bargain, and I want the guinea accordingly."

Defendant.—"Now, docthor, by vartue of your oath, answer this: 'Did you cure my wife?'"

Doctor.—"No; she's dead. You know that."

Defendant.—"Then, docthor, by vartue of your oath, answer this: 'Did you kill my wife?'"

Doctor.—"No; she died of her illness."

Defendant (triumphantly, to the Bench).—"Your worship, see this. You heard him tell our bargain; it was to kill or cure. By vartue of his oath, he done neither, and yet he axes his fee!"

DOESN'T COST ANYTHING.—Totling.—Why do you people borrow trouble so much?"

DIMLING.—"Because it isn't necessary to put up any collateral."

